# Brain basis of self: self-organization and lessons from dreaming

**DOI:** 10.3389/fpsyg.2013.00408

**Published:** 2013-07-16

**Authors:** David Kahn

**Affiliations:** Department of Psychiatry, Harvard Medical SchoolBoston, MA, USA

**Keywords:** self-organization, dreaming, brain lesions, self, consciousness

## Abstract

Through dreaming, a different facet of the self is created as a result of a self-organizing process in the brain. Self-organization in biological systems often happens as an answer to an environmental change for which the existing system cannot cope; self-organization creates a system that can cope in the newly changed environment. In dreaming, self-organization serves the function of organizing disparate memories into a dream since the dreamer herself is not able to control how individual memories become weaved into a dream. The self-organized dream provides, thereby, a wide repertoire of experiences; this expanded repertoire of experience results in an expansion of the self beyond that obtainable when awake. Since expression of the self is associated with activity in specific areas of the brain, the article also discusses the brain basis of the self by reviewing studies of brain injured patients, discussing brain imaging studies in normal brain functioning when focused, when daydreaming and when asleep and dreaming.

## Introduction

How do consciousness and a sense of self arise from processes occurring in the brain? Many authors have asked and suggested answers to this question including: Crick and Koch (1990), Rees and Frith (2007) on the neural correlates of consciousness; Hobson (2009), Långsjö et al. (2012) Dehaene and Changeux (2011), Revonsuo (2006) on a general theory of consciousness; Kahn and Gover (2010), Desseilles et al. (2011), Dang-Vu et al. (2005), Maquet et al. (2005), Hobson (2009), Domhoff (2001) on neural correlates of consciousness and theories of dreaming; Schwartz et al. (2005), Hobson et al. (1998), Ruby (2011) on a neuropsychological approach to dreaming consciousness.

One important contribution to a theory for the emergence of consciousness comes from Tononi and Edelman (Tononi and Edelman, 1998; Tononi, 2004). They argue that a certain degree of complexity must exist in order for consciousness to emerge. Consciousness will not emerge from a system of completely independent components, for example, a system of randomly acting molecules of a gas. At the other extreme, consciousness will not emerge from a completely ordered system like a crystal. In order for consciousness to emerge there has to be some degree of independence among the elements that make up the system and some degree of dependence or relationship between these elements. Tononi and Edelman further develop these ideas by showing that consciousness as information can be achieved for a specific degree of complexity. There is no information obtainable from a system of completely independently acting elements. Likewise, there is no information to be gained from a completely ordered self-contained system of tightly bound elements. From an information perspective, therefore, a system must have a degree of complexity in order for it to yield information: not too much independence and not too much dependence among its elements. Consciousness is a form of information and will emerge only in a sufficiently complex system of interacting elements, for example, neurons in the brain. The Tononi and Edelman theory provides a theory that explains why a sufficient amount of complexity must exist before consciousness can emerge.

The self-organization theory of consciousness introduced here in connection with dreaming consciousness adds a specific mechanism of self-organization to the information theory of consciousness and complexity. The self-organization theory postulates that consciousness can emerge when a threshold degree of complexity is reached among the cerebral processes in the brain. For self-organization to occur, there has to be a sufficient density of locally interacting neural elements that develop relationships. Self-organization occurs when a threshold of integration or relationship is reached.

### Self-organization and the self

As a result of a self-organizing process in the dreaming brain, a new self is created. By self is meant it is I who is doing the thinking and feeling and it is I who directs my actions. I have a body that is mine and not someone else's and I know where I am. The dream self too is the self who does the thinking and feeling and who is the protagonist in the unfolding dream. What emerges, however, is a unique self, a self that is created from the experiences of the dreamer, but put together in a unique way. The dream is put together via a self-organizing process.

A new dimension of the self occurs while dreaming because we are not actively controlling or directing the self. The ideas we have built up over a lifetime on who we are, what we will and will not do, and what we know is possible to do are mostly forgotten when we dream. The dream is not created by the will of the dreamer but by a coming together of memories, feelings, and new thoughts whose unsuspecting author is the dreaming brain. The dream self does not control how individual memories that were garnered over a lifetime become weaved into a dream. The dream self is a part of its own creation, and at the same time the dream self affects how the dream progresses.

### Limitations of the self-organization model

Self-organization, however, does not predict or explain the emergence of specific aspects of dreams, for example, the occurrence of social aspects of dreams [a dream is likely to have 4–5 characters participating in the dream including the dream self (Kahn et al., 2002)]. Content analysis provides much information on dream content (Hall and Van de Castle, 1966), for example, on such categories as the number of friendly or unfriendly interactions, the number of known vs. unknown characters, and a slew of many other important attributes of the dream self, such as the dream self appearing in a first person perspective vs. a field perspective watching the action unfold (Hall and Van de Castle, 1966; Domhoff, 2003). Self-organization theory of dream formation does not study the content of dreams; rather it provides a mechanism for how mental activity coalesces into a dream. For example, through the self-organizing process, people known and unknown to the dreamer can appear together in a dream; people in different time periods of the dreamer's life can appear together; and, equally important, self-organization can lead to a dream that is completely novel, even more so than a dream containing a complex combination of individuals, locations, and time periods. For example, some creative dreams are of things or people that do not exist in the dreamer's wake world. Examples of such creative dreams are given in Deirdre Barrett's book (Barrett, 2010) and Maquet and Ruby (2004). This author recently dreamed of a never before seen multi-dimensional and multi-colored wall-hanging that rhythmically undulated in and out of the walls of a person's apartment wall.

### Creation of the wake self

When awake, our sense of self is affected by a number of factors (Blanke and Metzinger, 2009; Ramanchandran, 2011). These include a feeling of *unity*; the sense that I am someone and that someone is I. I am not two or more different people, even if I may act that way on occasion, I am me, one body, I have this sense of unity even if I am made up of different parts, like heart, brain, bone, fingers. I have this sense of one me.

Another is the sense of *continuity*, the feeling that I have a history, I have had experiences, that there is continuity between who I was and who I am now. I may have changed my beliefs; I may have changed what I once considered important to now be unimportant. I may have changed my weight or my hairstyle over my lifetime, but I still have a feeling that it is my history, not someone else's.

Another is the sense of *embodiment*. I have a body and my body is different than someone else's body. My self is embedded in my body even if my body may have changed during my lifetime; it's my body that has changed. When this sense of embodiment is questioned as during an out-of-body experience (Blanke et al., 2004), or during an induced artificial body illusion (Slater et al., 2009), profound and often disturbing alterations in one's sense of self can ensue.

*Free will* is another attribute of the self; it is the feeling that I can do something if I want to. Contributing to a sense of self, in addition to the feeling of unity, continuity, embodiment is the dynamic sense that I am free to act, that I am not a puppet led by someone else.

*Self-location* is another aspect of the self; I know where I am located in space. Contributing to a sense of self is the knowledge that I am located somewhere in space; for example, I am sitting in a chair, standing by the podium, or lying in bed. And when I think about where I am in space, I have a sense that it is I who is occupying that position in space. In out-of-body experiences, for example, this sense of self is disrupted (Blanke and Metzinger, 2009).

Another aspect of the self is having a *first person perspective*. There are several degrees of first person perspective as discussed in Blanke and Metzinger (2009). For here, let it suffice to say that exhibiting a first person perspective contributes to a sense of self. Having a first person perspective translates to the knowledge and feeling that it is I who is thinking, it is I who is writing this sentence, and it is I who knows I am writing this sentence.

*Social embedding* is another attribute that often helps give us a sense of self. By social embedding is meant the feeling that I am a part of a larger community; that I am in relationship with and am able to recognize friends, family, and acquaintances. My sense of self is influenced by how I feel I fit in or not fit in with others, by a feeling of belonging or by a feeling of estrangement from the group.

### Dreaming and the self

Now we'll look at these aspects of the self, unity, continuity, embodiment, free will, self-location, first person perspective, and social embedding, as they are experienced during dreaming.

Unity exists in the dream state as well; when dreaming, we feel that it is I who is engaged in the action. The I who is engaged in the dream may differ from the I who am awake but the sense that there is an I who is experiencing exists in the dream.

The sense of continuity is different in the dream state. Unlike the feeling that continuity exists over one's lifetime when awake, when dreaming each dream can stand alone; the dream and even each dream segment can begin and end anywhere; in a way the self begins when the scene begins. Continuity of the self exists mainly only within the time period of the dream.

The sense of embodiment, that feeling that I have a body and my body is different than someone else's body, is much more plastic when dreaming because the dreamer may inhabit a much younger or older body and at times even one of a different gender.

The sense that one has free will, the ability to do something if one wants, is often lost in dreaming. At times, the dreamer feels like a participant in the unfolding action of the dream, rather than in control. For example, we may want to dial a phone number but cannot; we may want to apply the brakes in a fast moving vehicle, but cannot. Strictly speaking, however, the dream self still has free will since the dream self is trying to do what he or she wants to do even if unsuccessful.

Self-location is preserved for the dream self even though the dream self does not know that his or her physical body is in bed sleeping. The dream self believes its location to be wherever it is in the dream even if the location may be highly improbable.

A first person perspective, the feeling that it is I who sees and reacts, also occurs in dreaming, this most often as the protagonist in the action, though sometimes as a witness to the unfolding events of the dream. Thus, the first person perspective contributes to a sense of self also in dreaming.

Social embedding, the knowing that I am in relationship with and am able to recognize friends, family, and acquaintances, contributes to a sense of self when awake, but when dreaming, social relationships may change. For example, some dream characters may have a different name than the person it represents in one's wake life, or the dream character may be a blend of two or more known wake life characters. A common discrepancy was found to be in a dream character's behaviors compared to that of the person in one's wake life (Revonsuo and Tarkko, 2002; Kahn and Hobson, 2003, 2005; Kahn, 2007).

The most striking result was that in a large majority of cases the dreamer did not notice these discrepancies during the dream; they were noticed only upon waking from the dream. One reason for not noticing discrepancies during the dream is that the dreaming mind is unable to call upon external cues; our sense of self while dreaming occurs within the context and the reality of the dream. During dreaming we are caught up in the experience of the dream, because not only is our ability to utilize external memory sources greatly reduced but also our access to internal sources is selective (Fosse et al., 2003). Being caught up in the experience of the dream allows the dream self to expand beyond the episodic and autobiographical memories of the wake self.

### Dreaming and thinking

Our sense of self is also affected by how we think. While dreaming, we are mostly unable to engage our wake-state critical thinking because we rarely examine the premises of the dream. This is true even when wake-like thinking occurs within the dream (Kahn and Hobson, 2005). Our ability to think *within* a dream event is more or less preserved, but our thinking *about* the hallucinatory experience is mostly absent when dreaming; the dreamer only rarely questions how the world works while engaged in the dream. Thus, the dream self “allows” the improbable, implausible, and even the impossible to become a part of how it identifies itself.

## The self-organizing process

But, how is this altered dream self created? As stated in the first part of the Introduction, the dream self emerges through a self-organizing process. What is a self-organizing process, does it have wide application, and what are the reasons to believe that dreams emerge from a self-organizing process?

Self-organization provides a mechanism by which structure and behavior emerges from the interaction and the integration of its component parts without direction imposed from the outside (Seeley, 2002). To quote from a recent article (Sasai, 2013), *“Self-organization is the spontaneous formation of ordered patterns and structures from a population of elements (or individuals) that have no or minimal patterns (Yates et al., 1987; Camazine et al., 2001; Chuong and Richardson, 2009; Saetzler et al., 2011; Dobrescu and Purcarea, 2011). Self-organization is seen in a wide variety of non-living matter on earth, including molecular assembly in crystal formation and in nanotechnology (Whitesides and Boncheva, 2002), … This form of pattern formation is often associated with the concept of emergence, or the spontaneous appearance of an ordered property of the whole that cannot be explained by the sum of the complexity of its elements (Camazine et al., 2001).”*

And Camazine (2004) offers a reason for appreciating the importance of self-organization in biology: *“One of the mysteries of biology is how the enormous amount of morphogenic, physiological, and behavioral complexity of living organisms can be achieved with the limited amount of genetic information available within the genome. Self-organization is one solution to this problem.”*

### Examples of self-organization

Examples of self-organization abound both in nature and in the inorganic world. In the inorganic world, the laser is an example of the emergence of structure as a coherent beam of light. This occurs through the cooperative behavior of photons leading to the amplification and self-organization of a narrow frequency of photons, the laser beam. In the laser, all diverse light frequencies except one become “enslaved” by only one frequency, the frequency of the laser. The laser is an example where many frequencies of light interact but only one frequency remains after the different frequencies self-organize; the frequency of the laser light represents the winning coalition of frequencies (Haken, 1981, 1983, 1993; Kelso, 1995).

**Figure 1:**
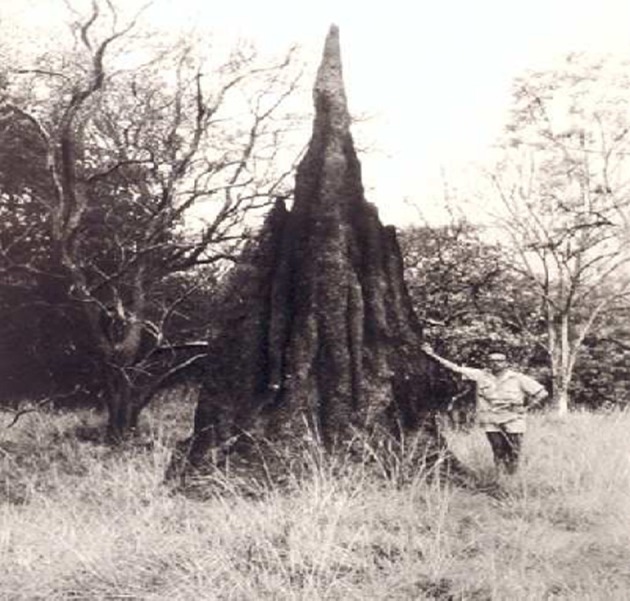
**A termite nest**. Taken from “An Introduction to Swarm Intelligence Issues” by Gianni Di Caro, Swarm-part-1.pdf. Individual termites will interact as they lay down pheromone. When a critical mass of termites and pheromone is reached, the termites gather together to begin building the termite nest. Individual termites through their interactions construct the termite nest without instruction from an architect.

In the organic world, the formation of a termite's nest (Figure 1) from local activity among many termites is an example of self-organization. A termite nest has an elaborate pillar and arch structure. A possible mechanism is suggested by Camazine (2004), *“Starting with a homogeneous, flat landscape, the random movements of the termites, and their dropping and picking up behavior leads to tiny surface irregularities which become the site of rising pillars. Once a pillar has emerged, this structure acts as a source of heterogeneity that modifies the actions of individual builders. The activity, in turn, creates new stimuli that trigger new building actions. Complexity unfolds progressively; increasingly diverse stimuli result from previous building activities, and facilitate the construction of ever more complex structures.”* The nest is the place where the termites live and reproduce; it is necessary for their survival.

Other examples of self-organization include the self-organization of bacterial colonies (Cho et al., 2007), the self-organization of birds into an efficient flight pattern, the self-organization of a raft by fire ants to enable them to cross a body of water (Mlot et al., 2011), and the self-organization of single cell amoeba into a multicellular organism called the dictyostelium discoideum that is capable of locomotion when a food source must be located (Marée and Hogeweg, 2001).

What is common in these examples is that an environmental change led to self-organization; an environmental change led to the existing system becoming unstable. This instability induced a re-organization from independent individual behavior to collective behavior that is able to meet the challenges of the changed environment. We shall come back to this idea when we discuss self-organization in the dreaming brain.

### Self-organization in the brain

Closer to home is the self-organization of a system of dynamically interacting neurons into a robust conscious state exhibiting emergent properties (Colangelo et al., 2004; Karsenti, 2008). As Singer (2009) states, *“The brain presents itself as a highly distributed, self-organizing system that self organizes and produces all those extraordinary phenomena that we as observers attribute to the person, the self.”*

The brain certainly has very many elements that interact, which is one condition for self-organization to occur. The brain consists of an enormous number of neurons (10^12^) each making on average 1000 connections with each other. As a result of local interactions within this large population of neurons, a competition forms between them until a subset of the winning synapses become established. This winning subset of neuronal synapses develops long-range coherence. This long-range coherence leads to growing complexity and to the formation of neural networks, then to a scaling up that eventually leads to consciousness, and to a sense of self.

### Self-organization in the dreaming brain

The self-organization model for the emergence of self is especially pertinent during dreaming when volitional control over access to memories is absent; memories come unbidden when dreaming. It is certainly the case that even when one has full volitional control as in the wake state, or partial volitional control as when lucid, self-organizing processes are taking place. But these self-organizing actions are mixed in with actions of the person him or herself who is able to consciously direct the action even while self-organizing processes such as internal generated patterns of behavior may be going on in the background. When lucid, there is both volitional and non-volitional control since when lucid one is in a hybrid state: one is aware that one is dreaming (Voss et al., 2009; Buzzi, 2011; Dresler et al., 2012). When lucid, both self-organization and dreamer-controlled action can occur. When not lucid, one is unaware that one is dreaming; the dreamer does not have control over the actions of the dream, and hence self-organizing processes is particularly germane to this non lucid state of dreaming.

As we saw, self-organization occurs when the environment changes so that a new system can emerge that is able to cope with the changed environment; in the case of dreaming it is the appearance of unsolicited thoughts and memories that lead to their self-organization into a dream. These unsolicited thoughts and memories will self-organize into a coherent whole (Kahn and Hobson, 1993). This is true whether the memories appear at random (Hobson, 1988) or stem from specific wake life memories and concerns. Since the unsolicited memories are self-organized, the dream that emerges is not under control of the dreamer; a new dimension of the self can, therefore, emerge in dreaming. As a consequence of this self-organization of different memories, dreaming allows the self to have experiences the wake self may not have dared to have.

The reason why any particular memory appears in the dreamer's mind is outside the scope of this paper because self-organization does not predict which memories will appear. Theories that do address specific content include continuity theory (Schredl and Hofmann, 2003; Schredl and Reinhard, 2009-2010); Jungian and Freudian theories, and the threat simulation theory (Valli et al., 2005). While self-organization does not address which specific dream elements appear in the dreamer's mind, the self-organizing process explains the emergence of a dream from the individual dream elements that do appear. And, as we have seen, self-organization has a function; it creates a new system capable of existing in a changed environment. For example, starvation and the necessity to find food led individual cells to self-organize into the mobile dictyostelium discoideum organism; the necessity to cross a body of water led individual ants to self-organize into a raft made up of their own bodies; and the need to sustain a colony of termites led individual termites to self-organize to build a termite nest. The human need to connect and make sense of unconnected or loosely connected dream elements will lead to their self-organization into a dream. The fact that a dream is formed from disparate dream elements suggests that a function of the self-organizing process may simply be to create a coherent story (the dream).

While the content of mental activity depends upon many things, as we have said, the self-organization process, which puts this content together into the dream, depends upon the environment. All self-organizing systems depend on the environment as we discussed in a previous section. The environmental change here occurs as we go from wake to the different stages of sleep. The brain's chemistry and specific regional activity changes as we enter the different stages of non-rapid eye movement (NREM) sleep and then again when we enter REM. The ability to integrate information in NREM is impaired due to a reorganization of large-scale networks into smaller independent modules (Bolya et al., 2012). There is also a time of night effect which affects brain activity and brain chemistry (Payne and Nadell, 2004). In other words, we suggest that one reason dreams can be so wildly different from one another is because of the different brain environments that can exist. The process of dream formation through a self-organizing process will yield different types of dreams in the different brain environments of NREM, REM, and time of night.

### Predictions of the model

Self-organization hypothesizes that a full-fledged dream appears. It is a model for how the different elements that appear in the dream are woven together to make the dream. In the language of physics, a phase transition occurs. All self-organized entities appear as a whole, spontaneously, our model claims that the dream appears as a whole spontaneously. The dream is formed from elements but these elements do not stand off by themselves once they have been organized into the dream. It is only when we are awake and ponder on the dream do we sometimes dissect the dream, some of us in order to find meaning, others to find function, others to look for relationship to everyday occurrences.

One prediction of the model is in agreement with the idea that dreams are a creation and not a replay of wake events and with the ideas of central image theory (Hartmann, 2008, 2010). The self-organization model predicts that a central story or theme will emerge from the interaction of elements. As Haken (1993) states it when discussing self-organization of the laser, one central frequency will enslave all the others. This idea here is that self-organization is a process by which the interacting elements compete with each other until a dominant one succeeds, in a sense, enslaving all other elements (Haken, 1993).

The protoconsciousness theory of Hobson (2009) postulates that brain activity from the fetus onward is necessary for the eventual development of consciousness. Brain activity may or may not be accompanied by dreams. For example, brain activity during REM in the fetus may not be associated with dreams. Mental activity in the form of dreams appears later, though exactly when is not specified by the theory. When it does appear, the protoconsciousness theory suggests that dreams may provide the ultimate simulation for coping successfully in one's wake life activities. Our self-organization model of how dreams are created from the elements of memory fragments can be considered an addendum to the protoconsciousness theory in that it suggests a way that dreams form from mental activity. This mental activity according to both theories is the result of brain activity. According to the protoconsciousness theory, this brain activity is a necessary precursor to the eventual development of consciousness.

While protoconsciousness theory does not directly address how the dream is created, the activation synthesis theory (Hobson and McCarley, 1977) postulates that random firings of brainstem neurons upon the forebrain yield dream formation. This theory addresses mainly the activation part and not the synthesis, that is, not how the dream elements are synthesized into a dream.

The cognitive theory of Domhoff (2001, 2003) and others does address dream formation by postulating that dreaming like any mental process is a cognitive process, and as such should be placed in the same category as thinking. Dreaming then is thinking while asleep. Dreams form, as do our thoughts when awake from memories, thoughts, feelings, and imagination. To this we simply add that it is important to take into account the different activation and modulation (neurochemistry) that affects thinking in the sleep compared to wake states. What the self-organization hypothesis adds is a mechanism by which a dream emerges from interacting dream elements.

Another hypothesis is Revonsuo's threat simulation theory (Valli et al., 2005) for the function of dreams. However, this theory does not directly address a mechanism for the coalescing of memory fragments into a dream.

## How the self is created

### Lessons from brain-injured patients

How might mental illnesses or abnormal brain function give us clues as to how the self is created in healthy people? First, we review several studies that were able to associate specific changes in how the self is expressed to damage in specific brain areas; second, we will review studies of normal brain functioning in different states of consciousness.

One change in the self that is caused by brain damage is known as alien hand syndrome where a person's hand seems to have a will of its own, acting without instruction from its owner. *In alien hand syndrome a person no longer feels the unity of the self because there seems to be two independent selves that are competing with each other.* The person with this malady, while perfectly able to direct his non-alien hand to do his or her bidding, the alien hand refuses to take orders. The alien hand acts as if it is independent of its owner; the hand acts on its own. It turns out that this alteration in the sense of unity can result after damage to the anterior cingulate area of the brain.

Another alteration in the sense of self that can result from brain damage is the out-of-body experience in which a person feels as if he or she has left their body. *In an out-of-body experience, one's sense of embodiment is questioned, and hence one questions whether they inhabit their own unique body.* There are various forms of out-of-body experiences, one of which is when the patient feels that they have left their body and are hovering over it, watching the body from above. This alteration in the sense of the self as an embodied being can result after brain trauma to the frontal parietal regions and the temporal parietal junction.

**Figure 2:**
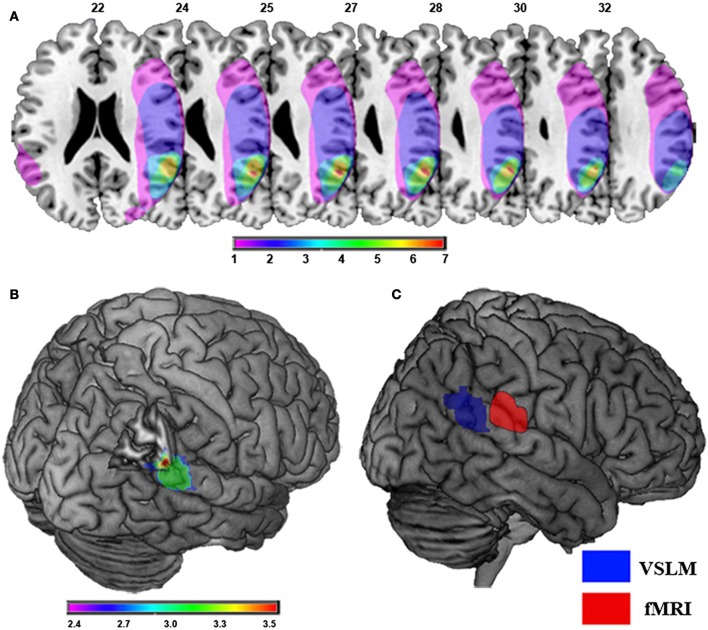
**Damage to the temporal parietal junction was responsible for the patient's out-of-body experience**. **(A)** Brain damage and results of lesion overlap analysis in nine patients with OBEs due to focal brain damage is shown. Maximal lesion overlap centers at the right TPJ at the angular gyrus (red). Overlap color code ranges from violet (one patient) to red (seven patients). Note that only one patient suffered from damage to the left TPJ. **(B)** Voxel-based lesion symptom mapping (VLSM) of focal brain damage leading to OBEs. The violet-to-red cluster shows the region that VLSM analysis associated statistically with OBEs as compared to control patients. The color-code indicates significant Z-Scores (*p* < 0.05; FDR-corrected) of the respective voxels showing maximal involvement of the right TPJ, including the right pSTG, angular gyrus, and middle temporal gyrus. **(C)** Self-location and the TPJ. Comparison between the area reflecting experimentally induced changes in self-location in healthy participants at the right TPJ using fMRI (red) and the area reflecting pathologically induced changes in self-location in neurological patients with OBEs using VLSM (blue). Taken from Ionta et al. (2011).

Out-of-body experience is directly related to damage to the temporal parietal junction area of the brain as indicated in Figure 2.

Another alteration in one's sense of self that is a result of brain damage is called the Capgras syndrome (Edelstyn and Oyebode, 1999). In Capgras syndrome, there is a misidentification conflict between what one sees and what one feels one is seeing. In this syndrome, a person may have no problem visually recognizing someone, but then insist that the person is an imposter, she's not who she looks like. This misidentification can occur even between close relatives, brother, sister, mother father, or spouse. *His sense of relationship and social embedding has been altered; he visually recognizes a person but refuses to believe his eyes. We normally use our relationships to help define ourselves. Since our sense of relationship is fractured, so too is our sense of self.* This condition can result after damage to emotional areas of the brain that results in a mismatch between emotion and visual identification.

While these examples are far from exhaustive, they do illustrate the close relationship between our sense of self and the integrity of our brains and help answer our question: how might mental illnesses or abnormal brain function give us clues as to how the self is created in healthy people?

### Learning from normal brain functioning

And how might normal brain functioning in different states of consciousness give us clues as to how the self is created in healthy people? We review studies that have identified brain area activity when thinking about one's self, when we daydream and when we are asleep and dream.

### Awake, focused, and daydreaming

Several midline cortical regions of the brain become active when we are thinking about our personality traits, when we are thinking about who we are. These midline cortical areas are the medial prefrontal cortex (MPFC), the posterior cingulate cortex (PCC), and the perigenual anterior cingulate cortex (PACC) (Parvizi et al., 2006; Andrews-Hanna et al., 2010; Ionta et al., 2011; Qin and Northoff, 2011).

Differences were found in brain activity when one was thinking about one self compared to thinking about someone else (Qin and Northoff, 2011). They found that there was activity in the anterior cingulate cortex for self-specific stimuli that is, thinking about one self, but not for non-self specific stimuli, that is, when thinking about someone else. This brain region specificity persisted even though an overlap in the midline cortical structures between self-specific and non-self specific stimuli exists. The overlaps are instructive in themselves.

One of these overlaps in self-specific and non-self specific thinking was the co-activation of the anterior insula cortex (AIC) and the posterior anterior cingulate cortex (PACC). The AIC in conjunction with the PACC contribute to humans being aware of themselves, others, and the environment (Craig, 2009). Thus, co-activation between the AIC and PACC seems to be important for creating a self who is in relation to other selves. Qin and Northoff (2011) suggest that this overlap of brain activity implies that the self cannot be regarded as being completely of internal origin, and cannot be regarded as coming purely from external stimuli.

To make this point even more cogent, it was found that activity in the PACC overlapped with activity in the default mode network (DMN) when self-specific stimuli were presented. The DMN is often considered a non-task driven state of consciousness that is associated with mind wandering or daydreaming. The DMN is attenuated only when engaged in goal-directed actions (Gusnard and Raichle, 2001). When we daydream we often recall autobiographical episodes; sometimes we mull over past encounters, and very often we find ourselves thinking about, and making plans for, the future. In short, we think about our self when we daydream (Spreng et al., 2008; Gruberger et al., 2011).

The overlap that occurs in all three regions, the PACC representing external stimuli, the AIC representing stimuli of internal origin, and the DMN representing the resting state when we daydream, emphasizes that the self cannot be regarded as being completely of internal origin, and cannot be regarded as coming purely from external stimuli. The self emerges from internal musings and external stimulation.

**Figure 3:**
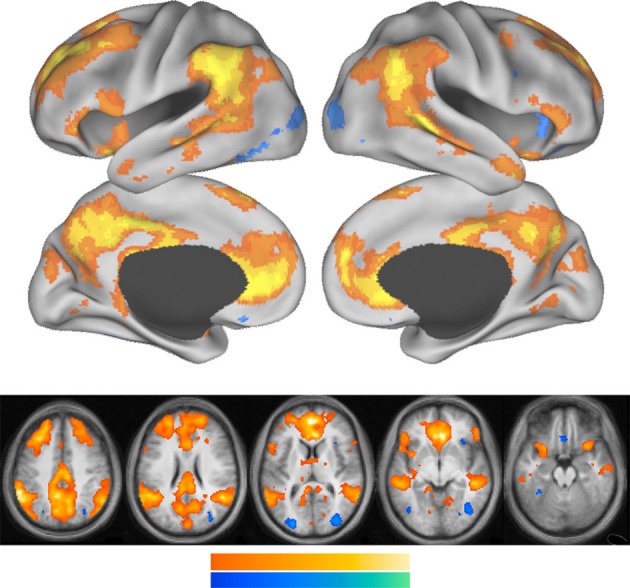
**This figure is from Andrews-Hanna et al. (2010)**. Whole-brain exploratory analyses were conducted using the main effect contrast of self trials vs. non-self control trials. Warm colors represent greater activation during self trials, whereas cool colors represent greater activation during non-self control trials. Increased activation during self trials was observed prominently in the PCC and the MPFC and temporal parietal junction.

Figure 3 shows that the core areas of the default network, the PCC and the anterior MPFC are activated when a person references him or her self. Other components of the default network also show increased activation during self-referencing.

### Asleep and dreaming

**Figure 4:**
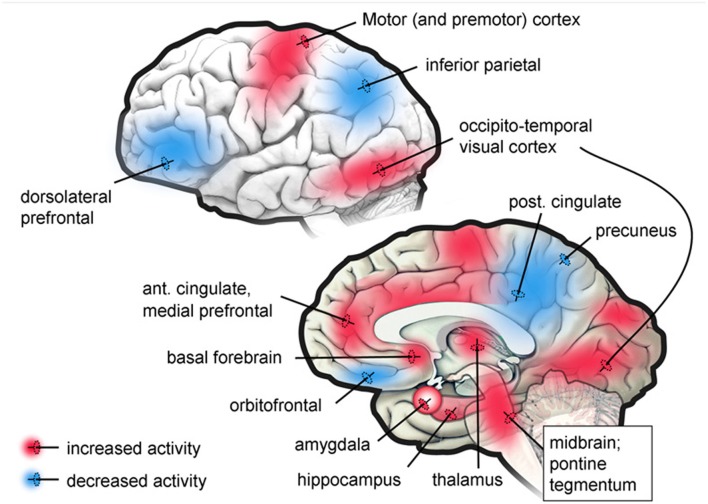
**Regions showing increased or decreased brain activity in the PET studies**. Taken from Desseilles et al. (2011).

Now, what about when we are not awake, focused, or daydreaming, but are asleep and dreaming? What is the brain basis for such changes in the self as a reduced ability to see implausibility, to identify people accurately and to remember that one is in bed sleeping? During the REM stage of sleep when dreaming is most vivid, there is a functional disconnection of the dorsal lateral prefrontal cortex with most of the rest of the brain, and a deactivation of large areas of the parietal cortex (Maquet et al., 1996, 2000; Braun et al., 1997, 1998; Maquet, 2000; Nofzinger et al., 2002). The functional disconnection of the prefrontal cortex significantly impairs the retrieval of episodic memory (Buckner and Koutstaal, 1998; Cabeza and Nyberg, 2000; Rees et al., 2002). Figure 4 shows PET study results in neural activity of specific brain regions when in the REM stage of sleep.

There are also changes in the chemical neuromodulation of the brain from aminergic to cholinergic when we move from wake to the different stages of sleep. The reduction and eventual absence of serotonin and norepinephrine in the forebrain as the subject goes from waking to NREM to REM sleep help account for the difficulty the dreamer has in noticing discrepancies between his dream and real-life characters, as mentioned previously.

Table 1 illustrates the relative amount of the important neurotransmitters, serotonin, norepinephrine and acetylcholine in the wake, NREM and REM sleep stages. The table shows that when awake and active the concentration of the neurotransmitters acetylcholine, norepinephrine, and serotonin is high. During the slow wave sleep (SWS) portion of NREM, the concentration of acetycholine decreases significantly while the concentration of norepinephrine and serotonin remains at the same level as in the quiet wake period. During REM, however, the concentration of acetycholine becomes very high while the concentration of norepinephrine and serotonin plummet to near zero levels.

**Table 1:** **Neuromodulation varies across the wake-sleep cycle**.

	**Active wake**	**Quiet wake**	**SWS**	**REM**
ACh	Very high++	High+	Low−	Very high+++
NE, 5-HT	Very high++	High+	High+	Low−

When awake the concentration of acetycholine (Ach) and serotonin (5-HT) and norepinephrine (NE) vary from very high (++) to high (+). When in slow wave sleep the concentration of acetycholine decreases (−) while that of serotonin and norepinephrine remains high (+); but in REM sleep the concentration of acetycholine sharply increases (+++) but that of serotonin and norepinephrine decreases (−).

Thus, quite remarkable and profound changes are occurring in the brain's chemistry throughout the night as the sleep stages go through their cycles.

These changes in the neurochemistry of the brain in the different stages of sleep affect the brain's mental activity and production of dreams and affect one's sense of self. The sense of self is affected because these neuromodulators affect cognitive function, mood, attention, the ability to retrieve memories, and the ability to pay attention. For example, the aminergic system plays a key role in maintaining vigilance, attention, and decision-making. The extreme change in brain chemistry whereby the aminergic system completely shuts down while the cholinergic system remains high contributes to the occurrence of hallucinatory images during dreaming and the reduced ability to recognize implausibility within the dream (Kahn and Gover, 2010). These are important factors that expand one's sense of self. One is freed from paying attention to logic and freed from making sense of one's surroundings. In dreaming, one is allowed to be in the experience no matter how unlikely it might appear to a wake person. One becomes open to whatever images, thoughts, and feelings emerge.

In addition to the important role played by the change in serotonin and norepinephrine levels in REM sleep on dreams and the dream self, as previously discussed, it is important to acknowledge the role of dopamine in the formation of the dream self. Since dopamine is related to goal-directed behavior and reward processing, it seems natural that dopamine changes play a role in the way the dream self conducts him or herself. The role of the mesolimbic dopaminergic system, reviewed in Perogamvros and Schwartz (2012), is active during sleep and undoubtedly contributes to dream genesis and content.

## Concluding remarks

How the self is expressed depends on brain functioning. In the unhealthy brain, depending on the patient's specific brain damage, the self may lose its sense of unity, continuity, embodiment or social embedding, resulting in an altered sense of self. As we saw in the Lessons from Brain-injured Patients section, *in alien hand syndrome a person no longer feels the unity of the self because there seem to be two independent selves that are competing with each other. In an out-of-body experience, one's sense of embodiment is questioned, and hence one questions whether they inhabit their own unique body. This alteration in the sense of the self as an embodied being can result after brain trauma to the frontal parietal regions and the temporal parietal junction.* In Capgras syndrome, *the patient visually recognizes someone but refuses to believe his eyes. We normally use our relationships to help define ourselves. Since our sense of relationship is fractured, so too is our sense of self. This condition can result after damage to emotional areas of the brain that results in a mismatch between emotion and visual identification.*

In a normal brain, the sense of self also depends on how the brain is functioning, which in turn depends on whether one is awake and focused, daydreaming or asleep and dreaming.

One major difference in how the self is expressed when awake and when asleep and dreaming is due to the changes in the neurochemistry and neural activity in the dreamer's brain that affect his or her sense of self. Another is that when dreaming it is not possible to conduct reality checks. When awake we might question the occurrence of something unusual but we rarely question the unusual when dreaming. And, while we can withdraw from the daydream, we are not always able to voluntarily wake ourselves to reenter the wake world. In the dream-state, we are caught up in the dream experience and cannot control entrances and exits. The dream self even when engaging in, and witnessing unusual behavior, is often unaware of anything being unusual. For example, the dream self may engage in behavior that the wake self would not; the dream self may be young or old; strong or weak, bold or shy. In this way dreaming reconstructs and expands the self. The dream self, like the dream itself, is an emergent creation arising out of an individual's experiences as well as from thoughts occurring when asleep and dreaming.

If a dream is recalled, one can explore aspects of the self by examining the dream. However, even when dreams are forgotten, the dream self has had the experience even if not recalled by the wake self.

Does the dream have a function? As arising from a self-organizing behavior, it does. Self-organization in biological systems is an answer to an environmental change for which the existing system cannot cope; self-organization creates a system that can cope in the newly changed environment. The self-organizing process in biological systems happens, as we have seen, as individuals engage in cooperative behavior to find food, cross a body of water or fly in an efficient flight pattern. In dreaming, the function of self-organization is to put together loosely connected memories into a dream. And, one function of the dream may be to insure we have a wide repertoire of experiences; this expanded repertoire of experience results in an expansion of the self beyond that obtainable when awake.

### Limitations of the model

One major shortcoming of the self-organization hypothesis of dream formation is the difficulty of proving the hypothesis. We have evidence that self-organization is a universal phenomenon occurring in nature whenever conditions are favorable. Favorable conditions include many locally interacting elements, a degree of independence but also a degree of relationship between the interacting elements (Tononi and Edelman, 1998), and a degree of non-linearity such that even small changes can produce a large change (at the tipping or bifurcation point). While we have argued that the brain meets the conditions for self-organization to occur, we were unable to demonstrate that the brain does indeed use this ubiquitous phenomenon to produce dreams. Other possible mechanisms for dream formation besides self-organization include a built in template for the formation of dream themes whereby elements come together from a built in template similar to Jung's idea of archetypal dream themes and the collective unconscious. With a built-in template, there is no need for self-organization. It would be nice to see an experiment that could distinguish between these two mechanisms of dream formation.

Finally, our hypothesis that a dream emerges when thoughts, feelings, and images spontaneously self-organize is not in contradiction to the ideas put forth by Nir and Tononi (2010) that dreams come more from our imagination than from our perceptions. In either case, these coalesce into a story that is the dream by a self-organizing process.

### Conflict of interest statement

The author declares that the research was conducted in the absence of any commercial or financial relationships that could be construed as a potential conflict of interest.
